# Sex Differences in Age-Associated Concentric Remodeling and Diastolic Dysfunction

**DOI:** 10.1016/j.jacadv.2026.102682

**Published:** 2026-04-01

**Authors:** Israel Gotsman, Donna R. Zwas, Andre Keren, Offer Amir, David Leibowitz

**Affiliations:** Heart Institute, Hadassah Medical Center and Faculty of Medicine, Hebrew University of Jerusalem, Jerusalem, Israel

**Keywords:** aging, diastolic function, echocardiography, sex

## Abstract

**Background:**

Diastolic dysfunction is a fundamental substrate in heart failure with preserved ejection fraction, with its modulation by age, sex, and cardiovascular risk factors under active investigation.

**Objectives:**

The purpose of this study was to determine the age onset of sex-related disparities in diastolic function to establish intrinsic sex differences in cardiac aging.

**Methods:**

We analyzed key echocardiographic parameters related to left ventricular geometric remodeling and diastolic function in a large cohort (N = 42,077; females/males: 18,476/23,601) using covariate-adjusted general linear models. A sensitivity analysis was performed on a subgroup (N = 14,250) free of comorbidities.

**Results:**

Significant age-by-sex interactions were found for all key parameters with covariate adjustment (*P* < 0.001), indicating that age-associated differences diverged significantly between sexes. Females demonstrated increased age-related interventricular septal thickening, left ventricular end-diastolic diameter decline, and a marked rise in relative wall thickness after age 60 (interaction F = 18.88) indicating a shift toward concentric remodeling. Diastolic functional decline also exhibited a sex-dependent age-associated pattern: e’ velocity decreased significantly and E/e’ ratio increased significantly in females from the sixth decade (F = 26.32). Older females also exhibited larger left atrial volume index and higher tricuspid regurgitation pressure gradients. A composite *z*-score of these parameters revealed significant sex-specific disparities, pinpointing the sixth decade as the age of marked divergence in diastolic function (F = 36.86). These patterns persisted in the subanalysis of individuals without comorbidities.

**Conclusions:**

The age-associated cardiac remodeling and reduced diastolic function in older females were independent of comorbidities, supporting intrinsic sex-specific differences in cardiac aging emerging after the menopausal transition. These findings support the development of precise, sex-specific prevention strategies.

Diastolic dysfunction plays a fundamental and central role in the pathophysiology of heart failure with preserved ejection fraction (HFpEF), which is a major and increasing public health challenge. This condition is a growing concern, particularly as the global population ages, and is exacerbated by the rising prevalence of key cardiovascular risk factors, including obesity and diabetes mellitus, which contribute significantly to the development and progression of diastolic dysfunction. A striking clinical observation is that older women are disproportionately affected by HFpEF, suggesting an intrinsic, sex-specific susceptibility that is potentially linked to fundamental differences in cardiac and vascular aging and physiological changes after menopause.^1^^,^^2^ Despite the clear epidemiological evidence, the precise interplay between age, sex, and risk factors in the development of diastolic dysfunction remains an active area of research.^3^ Determining when these sex disparities emerge across the lifespan, and whether they are primarily a consequence of higher exposure to comorbidities or reflective of an intrinsic biological vulnerability in the female heart or a combination of both, is essential for targeted prevention strategies. This study aims to address these gaps by leveraging a large, well-characterized cohort to delineate the age- and sex-specific patterns of change in key diastolic function parameters, investigate the independent effects of age, sex, and common risk factors on cardiac remodeling, and determine if the observed disparities in women are intrinsically related to aging/menopause or primarily driven by an accumulation of comorbidities.

## Methods

### Study design, participants, and data collection

All consecutive subjects who underwent echocardiography examinations recorded in the echocardiography database of a university-affiliated laboratory during a 12-year period (2011-2022) were retrieved for analysis. To ensure the independence of observations, only the first (index) echocardiogram meeting the inclusion criteria was included for each unique subject. Individuals aged ≥18 years were included in the current cohort. Inclusion required the availability of demographic/comorbidity data and diastolic tissue Doppler parameters (mitral e′ and E/e′). The subject selection process is summarized in Supplemental Figure 1. Medical diagnoses (International Classification of Diseases [ICD-9] codes) were retrieved from electronic medical records and were required to be present at or prior to the time of echocardiography. Renal dysfunction was defined by ICD codes for chronic kidney disease. Echocardiographic data including measurements of dimensions and calculations of volumes were performed according to standard recommendations of the American Society of Echocardiography and the European Association of Cardiovascular Imaging using 2-dimensional and/or linear, m-mode-guided measurements.^4^ Relative wall thickness (RWT) was calculated as: (2 × PWd)/LVEDD, where PWd is the left ventricular (LV) posterior wall thickness in diastole and LVEDD is the LV end-diastolic diameter. LV ejection fraction (EF) was measured at rest and was assessed either by the Simpson biplane method (18% of cohort) or estimated qualitatively by a trained cardiologist. Mitral e′ velocity and the E/e′ ratio were measured at the lateral mitral annulus to estimate ventricular relaxation and filling pressures. The lateral E/e' ratio was utilized as it was the most consistently documented diastolic parameter throughout the study period, reflecting its high technical feasibility and historical clinical use in our database. The institutional ethical committee for human studies of the university-affiliated hospital approved the study protocol. Informed consent was waived by the ethical committee as the study was observational and data were analyzed retrospectively.

### Statistical analysis

Baseline clinical characteristics were compared between sexes using analysis of variance (ANOVA) for continuous variables and chi-square tests for categorical variables. A General Linear Model framework was used to perform covariate-adjusted ANOVA on key echocardiographic parameters, with estimated marginal means calculated to delineate the independent effects of age and sex. The adjustment methodology standardized all comparisons to a healthy baseline by specifying custom covariate values within the model. The covariates included in the model were heart failure, hypertension, diabetes mellitus, ischemic heart disease, atrial fibrillation, renal dysfunction, obesity, and severe mitral regurgitation; for LVEF, the assessment method (qualitative vs quantitative) was additionally included. Each covariate was anchored to a value of zero, representing the complete absence of the disease or risk factor, for the calculation of all adjusted means presented in tables and figures. This approach ensured that the observed sex-specific differences in cardiac remodeling reflect intrinsic biological factors rather than differential disease burdens between the sexes. To evaluate the clinical magnitude of these findings, sex-specific adjusted mean differences with 95% CIs were calculated for each age decade (Supplemental Table 3). The assumption of homogeneity of variances was assessed using Levene’s test, and violations were observed in some analyses. However, the large sample size ensured that the F-tests remained robust to these violations, while approximate normality of the sampling distribution was supported by the Central Limit Theorem, permitting valid interpretation of the results. Missing data for echocardiographic parameters were low (1.3% to 8.3%), with completeness detailed in Supplemental Table 1. Patterns of missingness were evaluated using the SPSS Missing Value Analysis procedure. Little’s test indicated that data were not missing completely at random (chi-square = 1,916, df = 63, *P* < 0.001). Expectation–Maximization estimates closely approximated the observed distributions, suggesting that the low level of missingness did not materially distort distributional characteristics. Accordingly, an available-case analysis was applied for all separate covariate-adjusted General Linear Models to maximize statistical power.

To generate a single, robust measure of overall diastolic function and remodeling, a composite *z*-score was calculated by integrating 7 key echocardiographic parameters: interventricular septal thickness in diastole (IVSd), LVEDD, RWT, mitral valve (MV) lateral e' velocity, MV E/e' ratio, left atrial volume index (LAVI), and tricuspid regurgitation (TR) peak gradient. Each variable was individually standardized to a *z*-score using the study population's mean ± SD. Z-scores were directionally aligned so that higher values consistently reflected worse diastolic health. The composite *z*-score was computed as the unweighted arithmetic mean of these 7 scores. There was significant but moderate internal correlation between the parameters (Supplemental Table 2), demonstrating that they capture distinct but related aspects of cardiac aging without redundancy. This methodology provides a transparent, balanced metric of the cumulative remodeling burden, allowing for the characterization of age-related trends without the bias of arbitrary weight assignments. Because the composite *z*-score required complete data for all 7 components, it had higher missingness (∼19%). Multiple imputation (20 data sets, fully conditional specification) was performed as a sensitivity analysis to confirm robustness of the findings. In addition, a height-indexed sensitivity *z*-score was calculated using height-adjusted linear dimensions (IVSd and LVEDD) to ensure the observed age-sex interaction was independent of body stature. Finally, a parsimonious sensitivity *z*-score was calculated using only 1 representative parameter from each physiological domain: RWT (structural), E/e' ratio (hemodynamic), and TR peak gradient (downstream). This was performed to ensure that the identified age-sex interaction was not dependent on specific variable weighting or selection.

IVSd was selected as the primary measure of LV wall thickness rather than the PWd. This decision was made because both measures demonstrated a high degree of correlation, and including both in the analysis would introduce redundancy and overweight the wall thickness component in the composite *z*-score calculation (Supplemental Figure 2). Left ventricular mass index (LVMI) was considered but was not included in the composite *z*-score. Although the age-by-sex interaction was statistically significant for LVMI (Supplemental Figure 3) suggesting that LVMI increase was accelerated in women in older ages, its dependence on LVEDD, which undergoes an accelerated reduction in females, confounded the sex-specific structural differences; RWT was included as the appropriate and defining metric for concentric remodeling. A *P* value of <0.05 was considered statistically significant. SPSS version 17.0 for Windows (SPSS Inc) was used for the statistical analysis.

## Results

The cohort included 42,077 subjects. Table 1 presents the clinical and echocardiographic characteristics of the cohort stratified according to sex. We performed univariable and covariate-adjusted ANOVA on key echocardiographic parameters pertaining to diastolic function with the objective of providing a thorough assessment of the independent effects of age and sex on diastolic function (Table 2). Given the large sample size, the overall main effects and interaction patterns remained consistent between the unadjusted and adjusted models, with similar trends in mean values. We therefore present the covariate-adjusted findings, adjusted for potential confounding variables as detailed in the methods. The clinical magnitude of these sex-specific differences across all age decades is provided in Supplemental Table 3.

**Table 1:** Clinical Characteristics and Echocardiography Measurements of the Cohort, Stratified by Sex

	Female (n = 18,476)	Male (n = 23,601)	Total (N = 42,077)	*P* Value
Age (y)	61 ± 19	59 ± 18	60 ± 19	<0.001
Heart failure	3,352 (18)	4,636 (20)	7,988 (19)	<0.001
Hypertension	9,389 (51)	12,316 (52)	21,705 (52)	0.002
Diabetes mellitus	7,027 (27)	5,672 (33)	12,698 (30)	<0.001
Ischemic heart disease	3,589 (19)	9,967 (42)	13,556 (32)	<0.001
Atrial fibrillation	2,831 (15)	3,306 (14)	2,136 (15)	<0.001
Chronic renal failure	7,883 (16)	4,896 (21)	2,778 (18)	<0.001
Obesity	6,181 (17)	3,462 (15)	3,643 (16)	<0.001
Severe MR	440 (2)	515 (2)	955 (2)	0.19
LV EF (%)	60 ± 9	56 ± 11	58 ± 11	<0.001
LV IVSd (mm)	9.2 ± 2.8	10.0 ± 2.9	9.7 ± 2.9	<0.001
LV PWd (mm)	8.6 ± 2.3	9.3 ± 2.6	9.0 ± 2.5	<0.001
LV EDD (mm)	46 ± 6	50 ± 7	48 ± 7	<0.001
RWT	0.38 ± 0.10	0.38 ± 0.14	0.38 ± 0.13	<0.001
MV E/A	1.15 ± 0.77	1.19 ± 0.82	1.17 ± 0.80	<0.001
e’ lateral (cm/s)	9.9 ± 5.9	10.2 ± 5.6	10.0 ± 5.8	<0.001
MV E/e' (lateral)	9.3 ± 5.6	8.3 ± 4.5	8.7 ± 5.0	<0.001
LA volume index (mL/m^2^)	27 ± 11	27 ± 12	27 ± 12	<0.001
Peak RV-RA gradient (mm Hg)	28 ± 11	26 ± 10	27 ± 10	<0.001

Values are n (%) or mean ± SD. *P* values were calculated using Pearson’s chi-squared test for categorical variables and Student’s t-test for continuous variables.

EDD = end-diastolic diameter; IVSd = interventricular septal diameter in diastole; LA = left atrial; LV EF = left ventricular ejection fraction; MR = mitral regurgitation; MV = mitral valve; PWd = posterior wall thickness in diastole; RV–RA = right ventricular–right atrial; RWT = relative wall thickness.

**Table 2:** Univariable and Covariate-Adjusted Analyses of Variance of Age and Sex on Echocardiographic Parameters

	Unadjusted Analysis			Adjusted Analysis		
	Age	Sex	Age × Sex	Age	Sex	Age × Sex
LV geometric remodeling						
IVS diastolic thickness	545.47 (<0.001)	481.49 (<0.001)	19.00 (<0.001)	210.19 (<0.001)	451.42 (<0.001)	15.56 (<0.001)
LV end-diastolic diameter	45.61 (<0.001)	1,934.02 (<0.001)	2.997 (0.002)	147.09 (<0.001)	1,702.08 (<0.001)	5.11 (<0.001)
Relative wall thickness	234.02 (<0.001)	2.78 (0.095)	18.28 (<0.001)	153.14 (<0.001)	5.48 (0.019)	18.88 (<0.001)
LV diastolic function						
MV lateral e' velocity	1,066.52 (<0.001)	0.006 (0.938)	12.15 (<0.001)	632.34 (<0.001)	1.06 (0.304)	12.23 (<0.001)
MV E/e' ratio (lateral)	918.28 (<0.001)	166.63 (<0.001)	24.95 (<0.001)	289.45 (<0.001)	235.39 (<0.001)	26.32 (<0.001)
Downstream parameters						
Left atrial volume index	1,079.67 (<0.001)	7.12 (0.008)	8.23 (<0.001)	298.71 (<0.001)	2.66 (0.103)	5.61 (<0.001)
TR peak gradient	810.85 (<0.001)	51.42 (<0.001)	12.07 (<0.001)	281.76 (<0.001)	70.61 (<0.001)	11.39 (<0.001)
Combined parameters						
Composite *z*-score	1,978.419 (<0.001)	107.965 (<0.001)	39.468 (<0.001)	861.196 (<0.001)	126.988 (<0.001)	36.858 (<0.001)

Values are reported as F-statistics with corresponding *P* values. Unadjusted models included age, sex, and their interaction. Adjusted models additionally included heart failure, hypertension, diabetes mellitus, ischemic heart disease, atrial fibrillation, renal function, obesity, and mitral regurgitation as covariates. The composite *z*-score is an integrated measure combining the 7 individual parameters reported in the table: IVS diastolic thickness, LV end-diastolic diameter, relative wall thickness, MV lateral e' velocity, MV E/e' ratio, left atrial volume index, and TR peak gradient. Individual *z*-scores were directionally aligned so that a higher value consistently represents worse diastolic function before being averaged.

TR = tricuspid regurgitation; other abbreviations as in Table 1.

### Sex-specific left ventricular geometric remodeling

**Figure 1:**
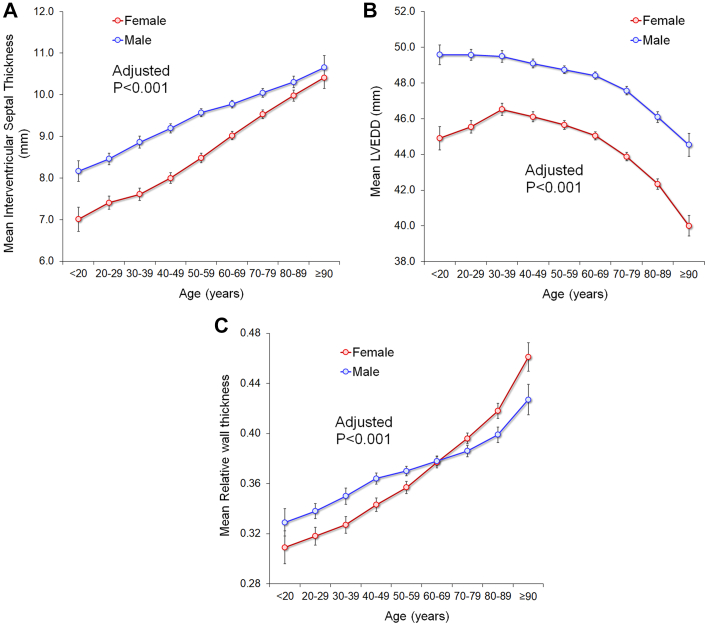
**Sex-Specific Left Ventricular Geometric Remodeling** Adjusted means plots illustrating the significant age-by-sex interaction effects on left ventricular geometric parameters. (A) Interventricular septal thickness in diastole. Interventricular septal thickness in diastole shows a narrowing disparity after the sixth decade, indicating amplified age-related thickening in older females. (B) Left ventricular end-diastolic diameter. The interaction reveals significant lower left ventricular end-diastolic diameter values in females after age 60, representing a more pronounced reduction in chamber size compared to males. (C) Relative wall thickness. Relative wall thickness age-associated patterns diverge sharply around age 60, with females exhibiting significantly higher relative wall thickness in later life.

We analyzed 3 LV geometric parameters: IVSd, LVEDD, and RWT. These key parameters provide insight into sex-specific LV geometric remodeling with age. After adjustment for covariates, all 3 parameters demonstrated significant age and sex interaction effects, highlighting critical differences in the pattern of cardiac remodeling of each sex with age (Figures 1A to 1C, Table 2). While males consistently had thicker IVSd, the sex disparity narrowed considerably from the sixth decade onward, indicating amplified age-related septal thickening in older females (Figure 1A). Similarly, males exhibited larger mean LVEDD, but the interaction effect revealed that females showed a significantly lower LVEDD after age 60, representing a more pronounced reduction in chamber size compared to males (Figure 1B). Furthermore, RWT was higher with age in both sexes but at very different rates of age-associated difference. The sex disparity in RWT values changed sharply around age 60; males had slightly higher RWT in younger age ranges, but females exhibited a significantly higher RWT in later life (Figure 1C). Taken together, these results point to pronounced concentric remodeling in females during a period consistent with the menopausal transition.

### Sex-specific left ventricular diastolic function

**Figure 2:**
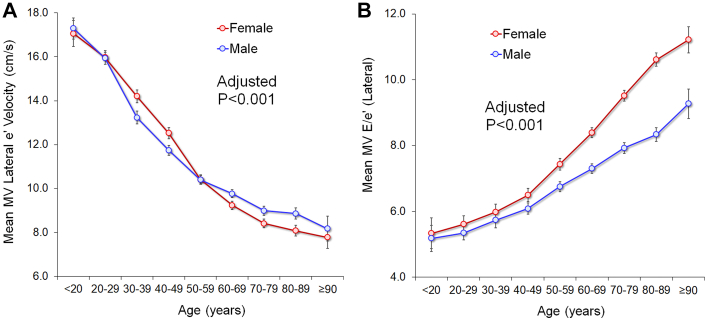
**Sex-Specific Left Ventricular Diastolic Function** Adjusted means plots illustrating the significant age-by-sex interaction effects on diastolic function. (A) Mitral valve lateral e' velocity. Velocity is significantly lower with age in both sexes, with a reversal in sex disparity occurring in the sixth decade. (B) Mitral E/e' (lateral) ratio. While females demonstrate consistently higher mean values, the sex-related disparity significantly widens beginning in the sixth decade of life. MV = mitral valve.

We analyzed 2 key parameters of left ventricular diastolic function: MV lateral e' velocity and the E/e' (lateral) ratio. These parameters characterize myocardial relaxation and LV filling pressures. After adjustment for covariates, both parameters demonstrated significant age and sex interaction effects, highlighting critical differences between males and females in diastolic function after age 60 (Figures 2A and 2B, Table 2). MV lateral e' velocity was significantly lower with advancing age in both sexes, with a reversal in sex disparity occurring in the sixth decade. Females demonstrated higher velocities between ages 30 and 50, but lower velocities than males in subsequent decades (Figure 2A). The E/e′ ratio was higher with advancing age in both sexes. While females consistently demonstrated higher mean values, the sex-related disparity in this ratio became statistically significant beginning in the sixth decade of life and progressively widened across subsequent older age groups (Figure 2B).

### Sex-specific left atrial and right-sided pressures

**Figure 3:**
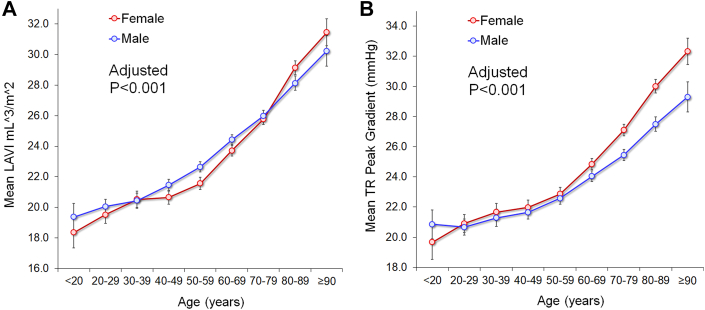
**Sex-Specific Left Atrial Volume and Right-Sided Pressures** Adjusted means plots illustrating the significant age-by-sex interaction effects on downstream pressures. (A) Left atrial volume index. The age-associated differences were more pronounced in females beginning in the sixth decade. This marked difference eventually led to a complete reversal of the sex disparity, with females exhibiting significantly larger left atrial volume index in the oldest age groups. (B) Tricuspid regurgitation pressure gradient. Females exhibit significantly higher values than males beyond the sixth decade. LAVI = left atrial volume index; TR = tricuspid regurgitation.

We assessed 2 additional parameters associated with downstream consequences of chronic left ventricular remodeling and diastolic dysfunction: the LAVI and the TR pressure gradient. After covariate adjustment, both parameters demonstrated significant age and sex interaction effects, revealing critical differences in the hemodynamic changes after age 60 (Figures 3A and 3B, Table 2). LAVI was significantly higher with advancing age in both sexes. While males had larger LAVI at younger ages, the age-associated differences steepened notably in females beginning in the sixth decade. This marked increase eventually led to a complete reversal of the sex disparity, with females exhibiting significantly larger LAVI in the oldest age groups (Figure 3A). The TR pressure gradient was significantly higher with advancing age, with females exhibiting significantly higher values than males beyond the sixth decade (Figure 3B).

### Left ventricular ejection fraction

LVEF also demonstrated a significant age-by-sex interaction (*P* < 0.001) (Supplemental Figure 4). The age-associated pattern of LVEF remained stable at a normal mean level in females through mid-life but was higher starting in the sixth decade. This high-normal LVEF in older females is consistent with the early phase of pronounced concentric remodeling and reduced LV cavity size observed in the female cohort. LVEF was not included in the composite score analysis of diastolic function.

### Sex-specific overall diastolic changes

To assess more rigorously when exactly the overall structural and functional changes related to diastolic function diverge between sexes, we performed a comprehensive analysis by combining the 7 key diastolic-related echocardiographic parameters. Integrated into a single standardized composite *z*-score, this approach revealed a highly significant age-by-sex interaction (*P* < 0.001) after adjustment for covariates (Central Illustration, Table 2). The composite scores demonstrated a steeper age-related slope in females compared with males, with females exhibiting progressively higher standardized values and clearly diverging from males beginning in the sixth decade. Sensitivity analyses with multiple imputation produced results consistent with complete-case analyses, confirming robustness. Furthermore, a sensitivity *z*-score utilizing height-indexed linear dimensions (IVSd and LVEDD) confirmed that this sex-specific divergence remains significant (*P* < 0.001) independent of body stature (Supplemental Figure 5). Finally, a parsimonious sensitivity model of the 3 physiological domains (RWT, E/e', and TR pressure gradient) yielded an identical age-by-sex interaction (F = 41.76, *P* < 0.001), reinforcing that the observed divergence is a robust, system-wide biological signal (Supplemental Figure 6).

**Central Illustration:**
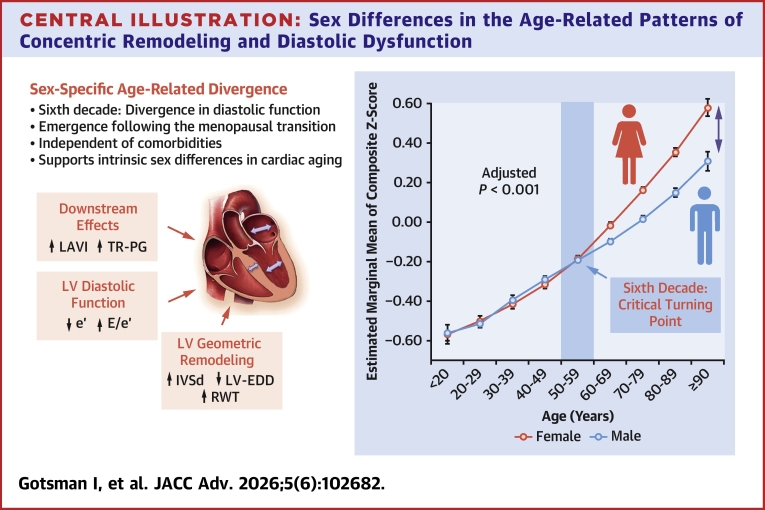
**Sex Differences in the Age-Related Patterns of Concentric Remodeling and Diastolic Dysfunction** (A) Sex-specific divergence in diastolic health: adjusted means plot. The plot shows the adjusted mean of the composite *z*-score combining key diastolic-related echocardiographic parameters, revealing a highly significant age-by-sex interaction. The age-associated differences in diastolic function were more pronounced in females (red), with the age-associated slopes clearly diverging from males (blue) in the sixth decade (50-59 years), which is identified as the age of significant divergence coinciding with the menopausal transition. (B) Study parameters and mechanism. The composite *z*-score integrated 7 key echocardiographic parameters, including: 1) Geometric measures (interventricular septal thickness in diastole, left ventricular end-diastolic diameter, relative wall thickness); 2) Functional measures (e', E/e'); and 3) Downstream indicators (left atrial volume index, tricuspid regurgitation-PG). The findings suggest that the pronounced cardiac remodeling and reduced diastolic function in older females are independent of comorbidities, supporting an intrinsic sex difference in cardiac aging that is temporally associated with hormonal changes associated with the menopause. IVSd = interventricular septal diameter in diastole; PG = pressure gradient; RWT = relative wall thickness; other abbreviations as in Figure 3.

### Sex-specific disparities in a subgroup without comorbidities

**Figure 4:**
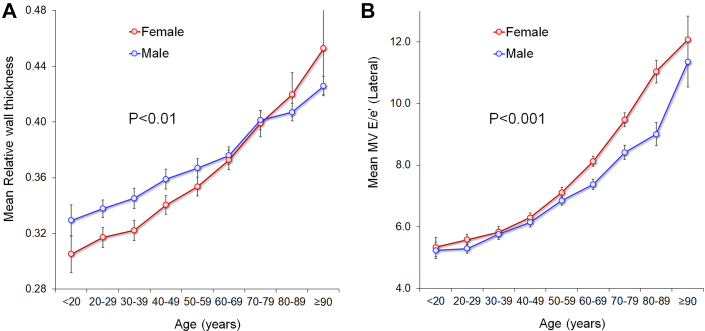
**Sex-Specific Disparities in a Subgroup Without Comorbidities** Adjusted means plots illustrating the persistent and statistically significant age-by-sex interaction effects for key parameters in the sensitivity subanalysis restricted to individuals without common cardiovascular risk factors or established disease. (A) Relative wall thickness. (B) Mitral E/e' (lateral) ratio. Abbreviation as in Figure 2.

To assess whether the observed sex-specific differences were influenced by common cardiovascular risk factors or established disease, we conducted a sensitivity subanalysis restricted to individuals without a history of heart failure, hypertension, diabetes mellitus, atrial fibrillation, renal dysfunction, ischemic heart disease, obesity, or mitral regurgitation (N = 14,250). The results of this analysis are presented in Table 3. The main effects of age, sex, and the critical age by sex interactions for key parameters, including RWT and the lateral MV E/e′ ratio, remained statistically significant and mirrored the patterns observed in the full cohort (Figure 4). These findings further suggest that the pronounced cardiac remodeling and reduced diastolic function seen in older females are independent of comorbid conditions and likely reflect intrinsic, sex-specific differences in cardiac aging.

**Table 3:** Sensitivity Analysis of Echocardiographic Parameters in a Healthy Subgroup

	Age (F-Stat, *P* Value)	Sex (F-Stat, *P* Value)	Age × Sex (F-Stat, *P* Value)
Relative wall thickness	79.83 (<0.001)	4.24 (0.039)	2.78 (0.004)
MV E/e' ratio (lateral)	367.88 (<0.001)	56.14 (<0.001)	9.76 (<0.001)

Values are reported as F-statistics with corresponding *P* values. The subgroup (N = 14,250) excluded individuals with a history of cardiovascular disease (heart failure, atrial fibrillation, ischemic heart disease, severe mitral regurgitation), metabolic disorders (hypertension, diabetes mellitus, obesity), or renal dysfunction.

Abbreviation as in Table 1.

## Discussion

In this large, population-based cohort, we observed clear sex-related patterns in LV remodeling and diastolic function with advancing age. After the sixth decade, women demonstrated pronounced concentric remodeling, characterized by septal thickening, reduced LV end-diastolic diameter, and increased RWT. These changes coincided with reduced diastolic function, reflected in lower e′ velocities and higher E/e′ ratios compared with men. Downstream consequences included larger left atrial volumes and higher TR pressure gradients, indicating a clinically relevant hemodynamic burden related to impaired diastolic function and increased filling pressures. The integration of these 7 individual parameters into a composite *z*-score effectively revealed this sex-specific divergence, pinpointing the steeper age-related differences in overall diastolic health in women beginning in the sixth decade. Importantly, these differences persisted after adjustment for comorbidities and were evident even among individuals without such conditions, suggesting intrinsic biological processes.^1^^,^^2^

These observations align with and extend previous echocardiographic studies documenting sex-specific differences in cardiac remodeling and diastolic function with advancing age. A longitudinal study from the Framingham Heart Study demonstrated accelerated concentric remodeling in older women.^5^ Sex differences in diastolic function were noted in healthy subjects^6^^,^^7^ and in the community,^8^ with women showing greater susceptibility to impaired diastolic function as they age. While prior studies highlighted a general greater susceptibility to impaired diastolic function with aging in women, our large, population-based cohort enabled us to accurately chart remodeling patterns across the lifespan. This study is among the first to explicitly identify the sixth decade as a pivotal divergence point in cardiac aging, characterized by pronounced concentric remodeling and reduced diastolic function in women. This temporal precision provides novel insight into HFpEF pathogenesis and defines a critical window for preventive strategies.

The divergence observed around age 60 corresponds with the completion of the menopausal transition and the onset of the postmenopausal period, as the mean age of natural menopause in the Israeli population is approximately 50 years. This temporal association potentially implicates hormonal changes in the sex-specific differences in cardiac aging. Declining estrogen has been suggested as an upstream factor that may amplify age-related processes, including maladaptive remodeling, microvascular dysfunction, and extracellular matrix changes leading to myocardial fibrosis. These effects are compounded by vascular dysfunction and stiffening also associated with hormonal changes with menopause,^9^ which increases late systolic load and further reduces compliance. In concert with women’s smaller ventricular cavities and concentric geometry, these mechanisms may explain the pronounced reduction in diastolic function and heighten susceptibility to elevated filling pressures and atrial dilation in older women.^2^^,^^10^, ^11^, ^12^, ^13^ This convergence provides a mechanistic basis for the disproportionate prevalence of HFpEF in older women.^14^^,^^15^ Taken together, these findings highlight how hormonal changes may amplify age-related remodeling to define the distinctive age-associated pattern of cardiac aging in women.

These mechanistic insights underscore the need for the development of sex-specific strategies in cardiovascular risk assessment and prevention.^16^, ^17^, ^18^ The pronounced reduction in diastolic function observed in women after age 60 highlights a sex-specific biological vulnerability. This may provide a mechanistic basis for the disproportionate prevalence of HFpEF in this population. HFpEF now accounts for more than half of all heart failure cases and disproportionately affects women, underscoring the urgency of timely intervention to slow progression and reduce disease burden. Preventive measures initiated around menopause, including strict blood pressure and diabetes management, lifestyle interventions and weight control, and therapies aimed at reducing myocardial fibrosis, may help mitigate disease development and lessen the burden of HFpEF. Furthermore, the persistence of these findings in individuals without comorbidities suggests that conventional risk-factor management alone may be insufficient. Biological vulnerability should be considered in clinical care, and hormonal influences may represent a potential target for early preventive strategies, although definitive clinical evidence is currently lacking.^3^

### Study limitations

Despite these important implications, several limitations warrant consideration. First, the cross-sectional design of this study precludes causal inference. While we observed clear age-associated patterns, these cross-sectional observations are inferred from population-level differences rather than confirmed longitudinal remodeling within individual subjects. Consequently, prospective longitudinal follow-up is required to confirm the precise temporal sequence of structural and functional decline and to account for potential cohort effects. Second, unmeasured confounding from factors such as physical activity, hormone therapy, genetic predisposition, socioeconomic status, or medication use may have contributed to the observed patterns. Furthermore, we lacked individual-level data on menopausal status and hormone replacement therapy. Therefore, the observed divergence in diastolic function remains a temporal association rather than a confirmed causal relationship driven by hormonal shifts. Third, echocardiographic data were derived from individuals referred to a university hospital, which may limit generalizability to broader populations. Consequently, our subgroup without comorbidities may be subject to referral bias, potentially limiting generalizability to the broader, asymptomatic population. Finally, measurement variability inherent to echocardiography should be taken into account, as technical and operator-dependent factors may introduce noise into the data. Incomplete or missing echocardiographic data sets may further limit the robustness of analyses, potentially introducing bias. Additionally, our reliance on lateral mitral annular velocities for consistency may be sensitive to regional wall motion abnormalities, annular calcification, or tethering, whereas averaged values are preferred by guidelines to mitigate such variations. Nonetheless, the large sample size, rigorous statistical adjustment, and consistency across comorbidity-free subgroups, together with sensitivity analyses using multiple imputation for missing data, strengthen confidence in the validity of our findings. Importantly, the convergence of multiple echocardiographic parameters on the sixth decade as a pivotal point of change underscores the robustness of this observation.

## Conclusions

This study demonstrates that women have pronounced concentric remodeling and reduced diastolic function after age 60 compared to men, independent of comorbidities. These findings highlight fundamental biological differences in cardiac aging and may provide a mechanistic basis for the higher prevalence of HFpEF in older women. Importantly, they underscore the need for the development of sex-specific prevention strategies, particularly around the menopausal transition.

Perspectives **COMPETENCY IN MEDICAL KNOWLEDGE:** Our study reveals that age-related cardiac remodeling is sex-specific, with a critical trajectory change for women beginning in the sixth decade of life. These findings enhance the understanding that older women exhibit unique patterns of accelerated concentric remodeling and diastolic decline independent of traditional comorbidities. Recognizing this distinct vulnerability is essential for practicing clinicians to implement earlier, sex-specific clinical actions. Providers should utilize this information to counsel female patients in their 50s and 60s regarding rigorous blood pressure management and lifestyle modifications. Such proactive strategies are aimed at mitigating identified remodeling patterns and preventing the later onset of heart failure with preserved ejection fraction (HFpEF).

**TRANSLATIONAL OUTLOOK:** Translating these observational findings into clinical practice requires addressing the current lack of evidence-based interventions specifically tailored to the post-menopausal trajectory. Future research should focus on the underlying biological mechanisms, including the role of hormonal shifts and their interaction with genetic factors. Large-scale prospective clinical trials are necessary to evaluate the efficacy of novel therapies targeting myocardial fibrosis and aortic stiffness in middle-aged women. Additionally, the development of non-invasive, serum-based or imaging biomarkers to predict individual susceptibility will facilitate personalized preventative medicine and reduce the long-term risk of heart failure in the female population.

## Funding support and author disclosures

The authors have reported that they have no relationships relevant to the contents of this paper to disclose.
