# Supplementary material for: Sex Differences in Age-Associated Concentric Remodeling and Diastolic Dysfunction
Source: JACC Adv. 2026 Apr 1;5(6):102682. doi: 10.1016/j.jacadv.2026.102682 (PMC13309338; doi:10.1016/j.jacadv.2026.102682)
Supplement: Supplemental_Material [file mmc1.docx]

Supplemental Table 1. Completeness of echocardiographic parameters

| Variable | Available N | % Missing |
| --- | --- | --- |
| LV EF (%) | 39,642 | 5.8 |
| LV IVSd (mm) | 39,491 | 6.1 |
| LV PWd (mm) | 38,839 | 7.7 |
| LV EDD (mm) | 39,955 | 5.0 |
| RWT (%) | 38,664 | 8.1 |
| MV E/A | 41,530 | 1.3 |
| e′ lateral (m/s) | 42,077 | 0 |
| MV E/e′ (lateral) | 42,077 | 0 |
| LA volume index (mL/m²) | 39,648 | 5.8 |
| Peak RV–RA gradient (mmHg) | 38,572 | 8.3 |

Total cohort N=42,077. Missingness across parameters ranged from 0–8.3%.

Supplemental Table 2. Pearson Correlation Matrix of the Composite Z-score Parameters

| Parameter | IVSd | LVEDD | RWT | e' | E/e' | LAVI | TRPG |
| --- | --- | --- | --- | --- | --- | --- | --- |
| IVSd | 1 |  |  |  |  |  |  |
| LVEDD | -0.11** | 1 |  |  |  |  |  |
| RWT | 0.45** | -0.43** | 1 |  |  |  |  |
| e' | -0.14** | -0.01* | -0.11** | 1 |  |  |  |
| E/e' | 0.27** | 0.04** | 0.16** | -0.33** | 1 |  |  |
| LAVI | 0.25** | 0.24** | 0.09** | -0.14** | 0.42** | 1 |  |
| TRPG | 0.15** | 0.09** | 0.08** | -0.11** | 0.36** | 0.43** | 1 |

* p < 0.05, ** p < 0.01.

IVSd, interventricular septal end-diastolic thickness; LVEDD, left ventricular end-diastolic diameter; RWT, relative wall thickness; e', lateral mitral annular early diastolic velocity; E/e', ratio of early transmitral flow velocity to early diastolic mitral annular velocity; LAVI, left atrial volume index; TRPG, tricuspid regurgitation peak gradient.

This matrix demonstrates the internal consistency of the composite Z-score. While each parameter provides distinct physiological information (as indicated by moderate correlation coefficients), all parameters are significantly correlated, justifying their integration into a single unweighted metric of global cardiac aging.

Supplemental Table 3: Magnitude of Sex-Specific Differences Across the Lifespan

| Age Group (years) | IVSd (mm) | LVEDD (mm) | RWT (Ratio) | Lateral e' (cm/s) | MV E/e' (Ratio) | LAVI (mL/m²) | TRPG (mmHg) |
| --- | --- | --- | --- | --- | --- | --- | --- |
| <20 | -1.16 (-1.55,-0.77) | -4.67 (-5.53,-3.81) | -0.02 (-0.03,-0.01) | -0.24 (-1.00,0.52) | +0.15 (-0.46,0.76) | -1.01 (-2.35,0.33) | -1.20 (-2.68,0.28) |
| 20-29 | -1.05 (-1.26,-0.84) | -4.03 (-4.50,-3.56) | -0.02 (-0.03,-0.01) | +0.04 (-0.37,0.45) | +0.26 (-0.07,0.59) | -0.54 (-1.27,0.19) | +0.24 (-0.56,1.04) |
| 30-39 | -1.25 (-1.46,-1.04) | -2.97 (-3.44,-2.50) | -0.02 (-0.03,-0.01) | +0.97 (+0.56,1.38) | +0.25 (-0.08,0.58) | +0.09 (-0.64,0.82) | +0.39 (-0.42,1.20) |
| 40-49 | -1.20 (-1.37,-1.03) | -2.98 (-3.36,-2.60) | -0.02 (-0.03,-0.01) | +0.79 (+0.45,1.13) | +0.41 (+0.14,0.68) | -0.80 (-1.40,-0.20) | +0.33 (-0.34,1.00) |
| 50-59 | -1.09 (-1.24,-0.94) | -3.09 (-3.41,-2.77) | -0.01 (-0.02,0.00) | +0.01 (-0.28,0.30) | +0.68 (+0.45,0.91) | -1.08 (-1.60,-0.56) | +0.30 (-0.27,0.87) |
| 60-69 | -0.76 (-0.89,-0.63) | -3.37 (-3.67,-3.07) | -0.00 (-0.01,0.01) | -0.53 (-0.79,-0.27) | +1.09 (+0.88,1.30) | -0.71 (-1.18,-0.24) | +0.81 (+0.31,1.31) |
| 70-79 | -0.52 (-0.67,-0.37) | -3.69 (-4.02,-3.36) | +0.01 (+0.00,0.02) | -0.58 (-0.86,-0.30) | +1.59 (+1.36,1.82) | -0.21 (-0.73,0.31) | +1.65 (+1.11,2.19) |
| 80-89 | -0.33 (-0.52,-0.14) | -3.75 (-4.16,-3.34) | +0.02 (+0.01,0.03) | -0.78 (-1.14,-0.42) | +2.27 (+1.98,2.56) | +1.01 (+0.37,1.65) | +2.51 (+1.86,3.16) |
| ≥90 | -0.24 (-0.63,0.15) | -4.53 (-5.40,-3.66) | +0.03 (+0.02,0.04) | -0.40 (-1.16,0.36) | +1.95 (+1.35,2.55) | +1.22 (-0.12,2.56) | +3.02 (+1.69,4.35) |

Data presented as Adjusted Mean Difference (Female-Male) and 95% Confidence Interval.

IVSd, Interventricular Septal Thickness; LVEDD, Left Ventricular End-Diastolic Dimension; RWT, Relative Wall Thickness; Lateral e', Lateral Mitral Annular Early Diastolic Velocity; MV E/e', Ratio of Early Transmitral Flow Velocity to Early Diastolic Mitral Annular Velocity; LAVI, Left Atrial Volume Index; TRPG, Tricuspid Regurgitation Peak Gradient; CI, Confidence Interval.

**Supplemental Figure 1. Study Flow Diagram**

Selection of the study cohort from 100,907 echocardiographic examinations (2011-2022). To ensure statistical independence, repeat examinations were excluded, retaining only the index study for each unique subject. Following the exclusion of pediatric cases and those with missing clinical or diastolic Doppler data, the final analytical cohort consisted of 42,077 unique subjects.

**Supplemental Figure 2: Comparison of Age by Sex Interactions for Wall Thickness Measures.**

**(A)** Estimated mean Interventricular Septal (IVS) diastolic thickness adjusted means plots across age, stratified by sex. **(B)** Estimated mean Posterior Wall (PW) diastolic thickness adjusted means plots across age, stratified by sex. Both had a significant age by sex interaction. The highly similar trajectory of the PW trend (Panel B) compared to the IVS trend (Panel A) shows the measures are highly correlated.

**Supplemental Figure 3**: **Age by Sex Interactions of Left Ventricular Mass Index (LVMI)**.

The covariate-adjusted mean estimated plot for LVMI. The age by sex interaction was statistically significant, reflecting a more pronounced increase in LVMI in older females. The steeper age-related reduction in LVEDD in females complicates the interpretation of sex-specific structural differences, as LVMI calculation is inherently dependent on LVEDD.

**Supplemental Figure 4. Age- and Sex-Specific** **patterns of Left Ventricular Ejection Fraction (LVEF).**

Covariate-adjusted mean LVEF remains stable in females through mid-life but shows a significant increase starting in the sixth decade, diverging from the slope observed in males. This pattern aligns with early pronounced concentric remodeling and reduced LV cavity size in older females.

**Supplemental Figure 5. Sensitivity Analysis of Height-Indexed Composite Z-Score.** Pooled estimated marginal means demonstrating the sex-specific, age-related remodeling and diastolic patterns when linear dimensions (LVEDD and IVSd) are indexed to height. The female age-related trend (red) reflects a steeper shift toward a concentric remodeling and diastolic dysfunction phenotype, significantly surpassing the male profile (blue) beginning in the sixth decade (P < 0.001 for age-sex interaction). Error bars represent standard error.

**Supplemental Figure 6. Sensitivity Analysis of a Parsimonious Three-Domain Composite Z-Score.**

Estimated marginal means for a reduced composite Z-score incorporating one representative parameter from each physiological domain: Relative Wall Thickness (Structural), E/e' (Hemodynamic), and Tricuspid regurgitation pressure gradient (Downstream). The age-related trend remains nearly identical to the primary 7-parameter model, with a highly significant age-sex interaction (F = 41.76, P < 0.001) and a clear divergence in the sixth decade. This supports that the observed findings are robust to variable selection and represent a consistent, system-wide biological signal.


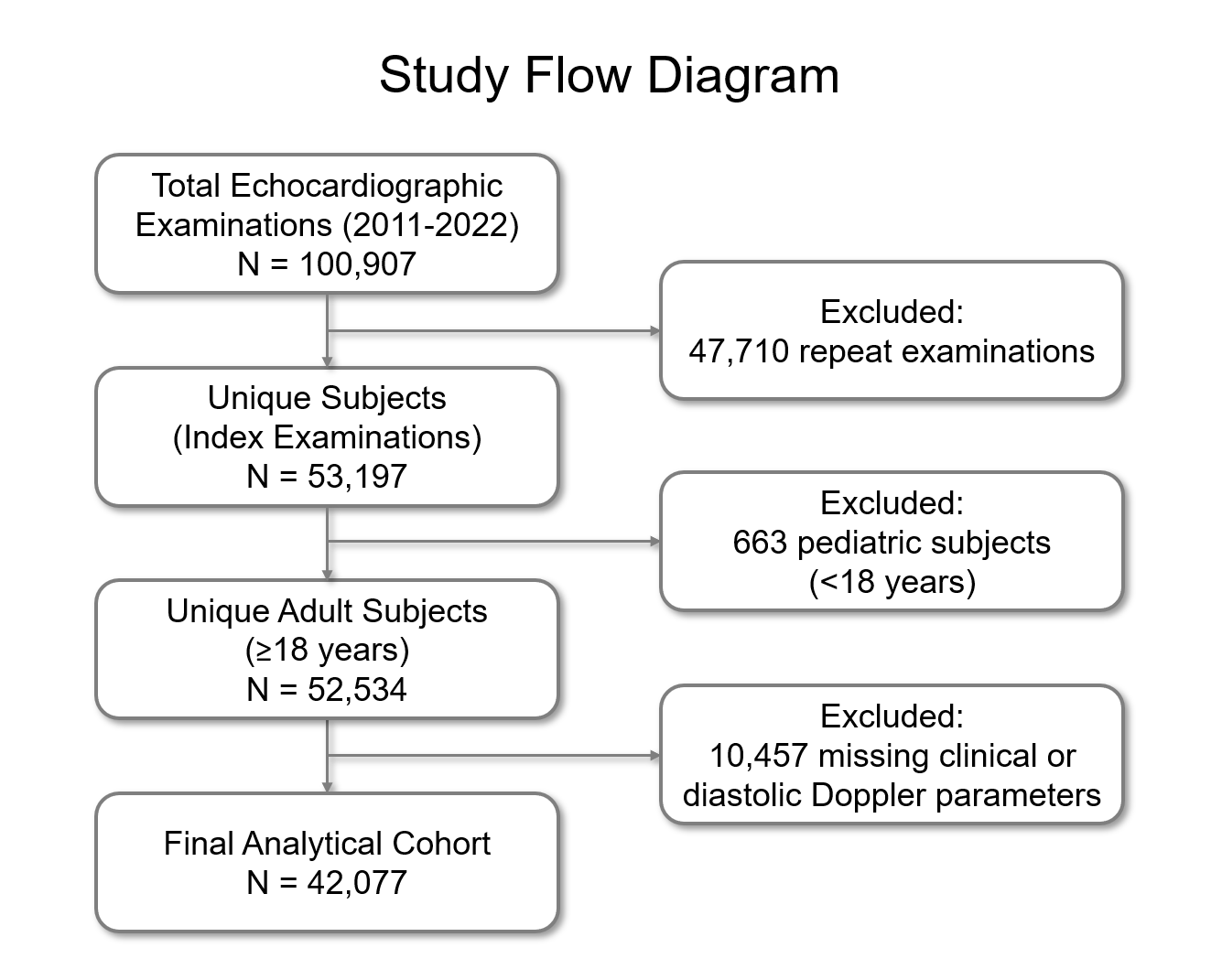


Supplemental Figure 1.


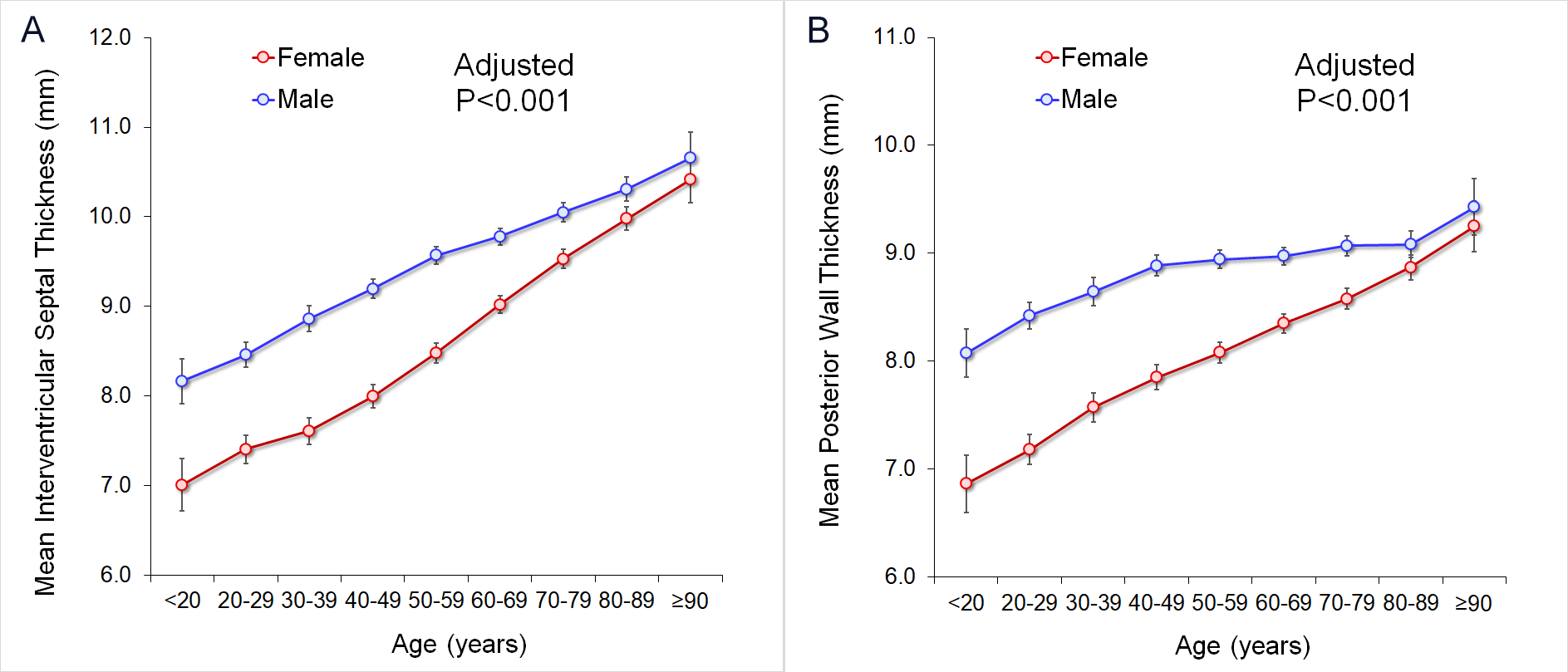


Supplemental Figure 2.


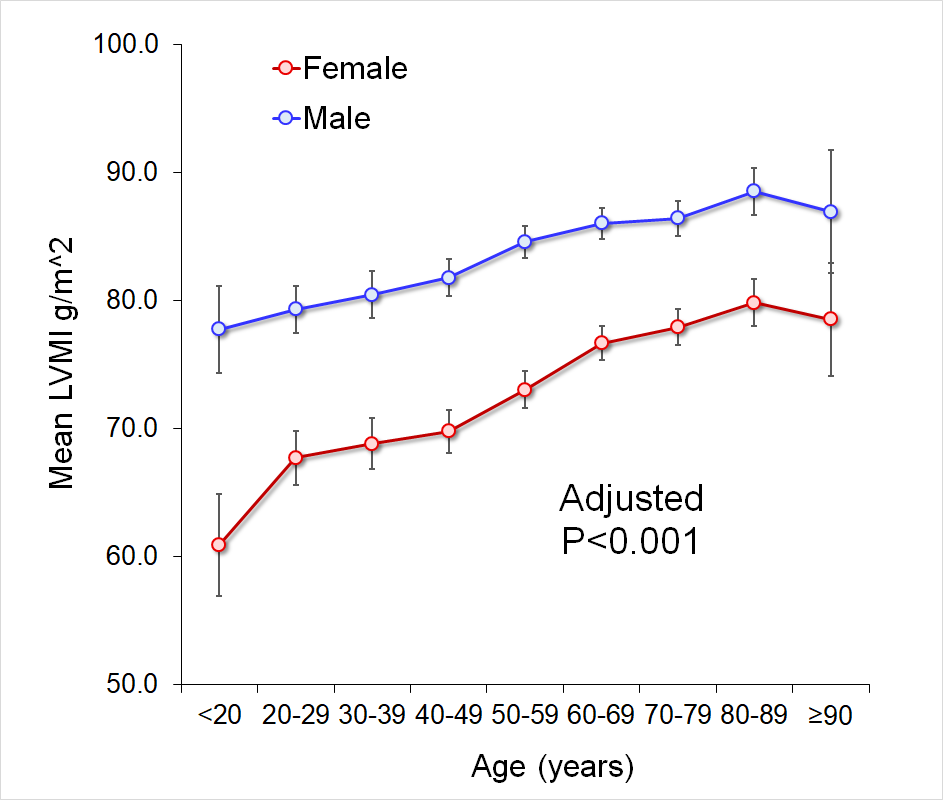


Supplemental Figure 3.


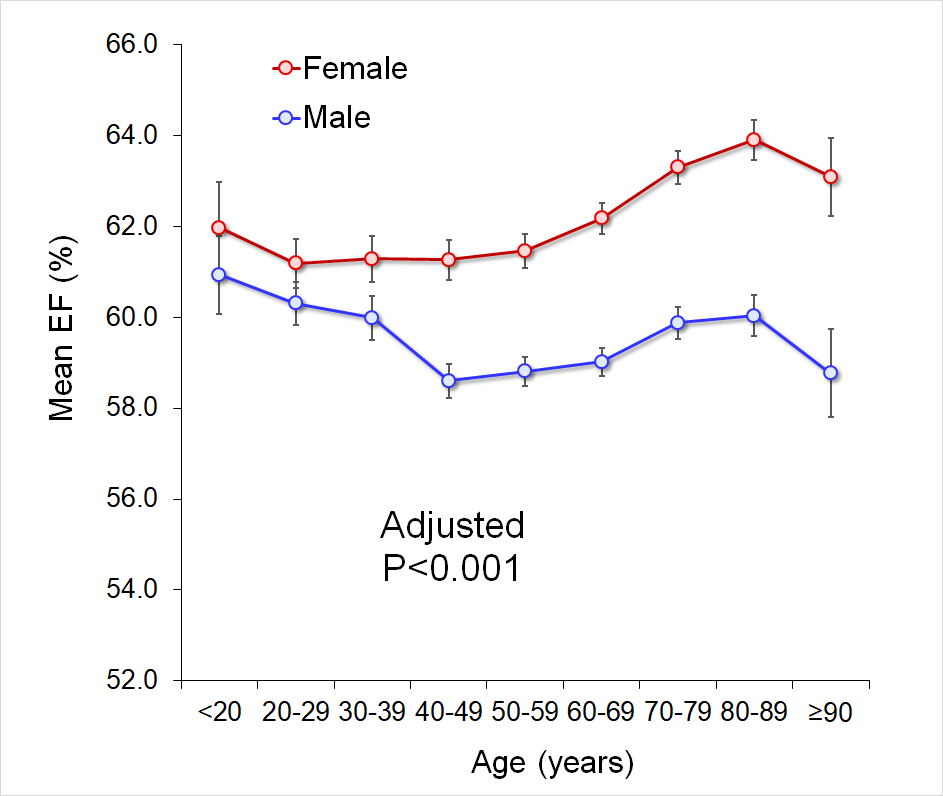


Supplemental Figure 4.


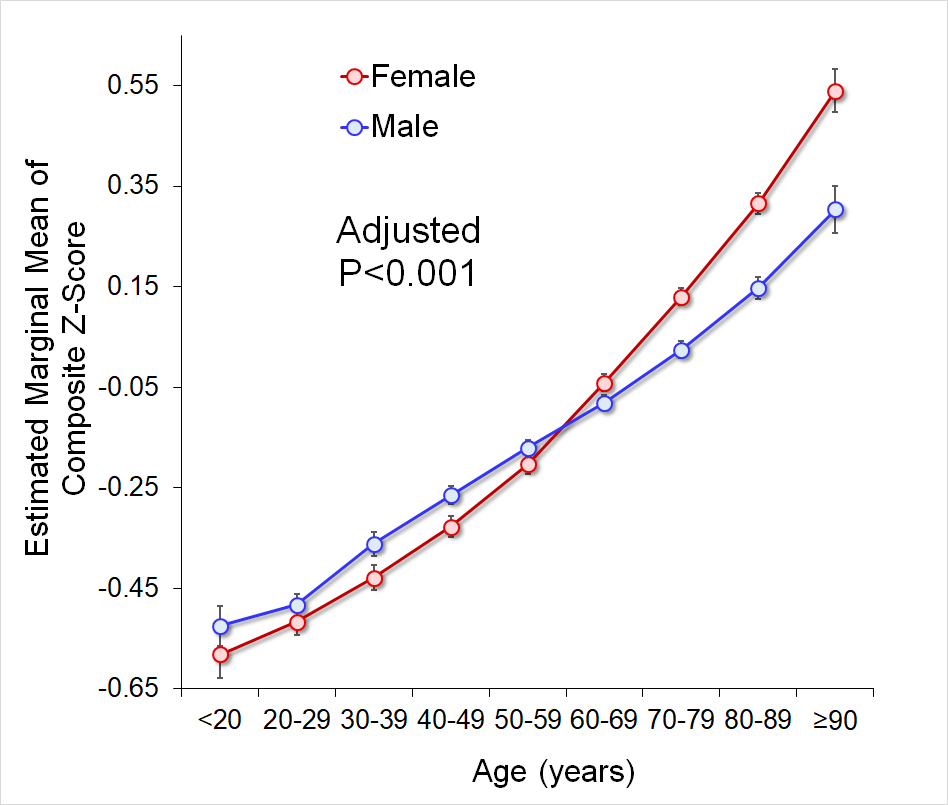


Supplemental Figure 5.


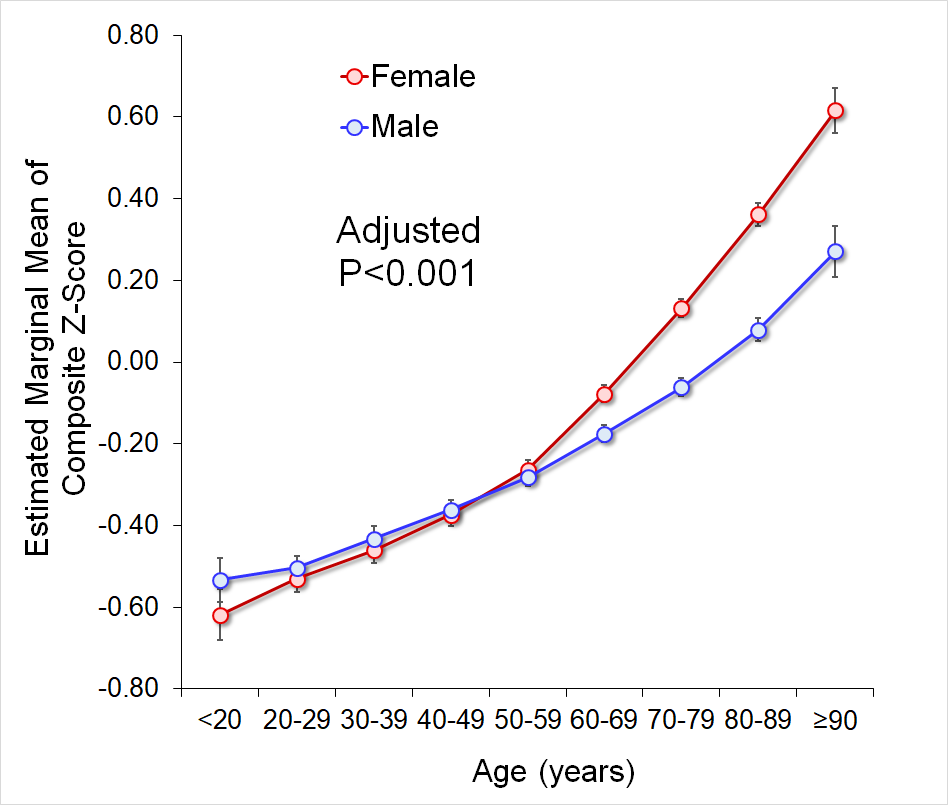


Supplemental Figure 6.
